# 3D-Printed Patient-Specific Models of the Aortic Arch for Advanced Visualization of Complex Neurointerventional Cases

**DOI:** 10.31486/toj.24.0124

**Published:** 2025

**Authors:** Smruti Mahapatra, Vishal N. Bhimarasetty, Abdul Rahim, Colin N. Curtis, Paul Gulotta, Korak Sarkar

**Affiliations:** ^1^ Department of Neurosurgery, Tulane University School of Medicine, New Orleans, LA; ^2^ Neurosciences BioDesign Lab, Ochsner Clinic Foundation, New Orleans, LA; ^3^ The University of Queensland Medical School, Ochsner Clinical School, New Orleans, LA; ^4^ Department of Interventional Radiology, Ochsner Clinic Foundation, New Orleans, LA; ^5^ Ochsner Neuroscience Institute, Ochsner Clinic Foundation, New Orleans, LA

**Keywords:** *Anatomic variation*, *cerebrovascular disorders*, *models–anatomic*, *neuroanatomy*, *patient-specific modeling*, *printing–three-dimensional*, *stroke*

## Abstract

**Background:**

Cerebrovascular disease is a leading cause of death and disability worldwide, and endovascular therapies have become a mainstay of treatment for ischemic stroke. However, tortuous anatomy, particularly of the aortic arch, presents formidable treatment challenges by impeding access to intracranial circulation and thus affecting clinical outcomes.

**Methods:**

To better understand the challenges of tortuous anatomy, we fabricated 3D-printed models of the aortic arch and major branch vessels based on the imaging of 4 patients.

**Results:**

These patient-specific models were realistic representations of the intricate vascular pathways and provided enhanced visualization of the complex vascular structures. The measured diameters of the 3D-fabricated models closely matched the values reported in the literature, confirming the physical accuracy of the models. Creating an individual anatomic model required an average of 4 hours of digital processing and 13.71 hours of 3D printing, with a materials cost of approximately $17.31.

**Conclusion:**

3D-printed patient-specific models used for neurointerventional training and preprocedural planning are a valuable tool for managing complex cerebrovascular anatomy. The advanced visualization provided by these models may enhance preparedness and potentially improve ischemic stroke treatment outcomes.

## INTRODUCTION

Cerebrovascular disease is a serious condition that results from occlusion (ischemia) or rupture (hemorrhage) of blood vessels supplying the brain. Ischemic strokes, intracerebral hemorrhage, subarachnoid hemorrhage, and vascular malformations are included in the cluster of cerebrovascular diseases.^1^ According to the 2019 Global Burden of Diseases, Injuries, and Risk Factors Study, stroke is the second-leading cause of death and the third-leading cause of death and disability combined.^2^ Further, the prevalence of cerebrovascular disease in younger age groups (<70 years) is increasing.^2,3^ Several risk factors are associated with cerebrovascular disease, chiefly smoking, hypertension, and alcohol intake.^4^

Time is a critical factor in minimizing damage to the affected tissue and preserving the remainder of the brain tissue; hence, prompt and accurate diagnosis of cerebrovascular disease is crucial. Modalities such as computed tomography angiography (CTA) and magnetic resonance imaging (MRI) of the brain are used to detect the presence, location, and severity of acute infarcts or hemorrhages.^5,6^ Newer imaging modalities such as vascular imaging and 3D rotational angiography are also used to locate lesions in the cerebral vasculature.^5^ Depending on the time window and the size of the occluded vessel, intravenous thrombolytic agents or endovascular thrombectomy are used to dissolve or remove the clot.^7^ Endovascular techniques typically involve inserting a guidewire through a femoral artery and advancing the guidewire through the intracranial circulation until it reaches the thrombus; then a device such as an aspiration catheter or stent retrieval tool is used to extract the clot.^8^ An angiogram confirms removal of the thrombus and recanalization of the vessel.^8^

The aortic arch is the central access point for cerebral endovascular procedures. Aortic arch variants are classified based on the vertical distance from the origin of the brachiocephalic artery to the top of the arch^9^:

- Type I – the distance is less than the diameter of the left common carotid artery.
- Type II – the distance is 1 to 2 times the diameter of the left common carotid artery.
- Type III – the vertical distance exceeds twice the left common carotid artery diameter.

Type III arches are associated with increased time requirements for manipulating the catheter during endovascular procedures.^9^ Tortuous vascular structures exacerbate the anomaly further, potentially leading to worse clinical outcomes given the time-sensitive nature of cerebrovascular disease.^10^

The advanced visualization provided by digital 3D models of patient-specific anatomy and 3D-printed models for rapid prototyping are emerging solutions to the challenges posed by aortic arch anatomy.^11-15^ Neurointerventionalists can use these models to visualize patient-specific anatomy and tailor their endovascular approach accordingly.^11-18^ Attendings and trainees can use 3D-printed models to practice complex procedures in a simulated environment.^12-18^ Advanced visualization and 3D-printed anatomic models can also be used for patient education.^18-20^

We selected 4 clinical cases in which abnormal patient anatomy created complications during endovascular thrombectomy. To better visualize and understand the challenges presented by these cases, we used 3D printing to retrospectively create patient-specific anatomic models of anomalous aortic arches and the major arteries originating from the arch.

## CLINICAL HISTORIES

**Figure 1.**
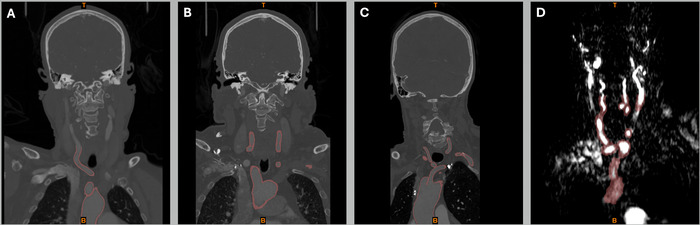
Coronal DICOM (Digital Imaging and Communications in Medicine) images of the aortic arch vasculature from 4 patients: (A) case 1, (B) case 2, (C) case 3, and (D) case 4. Each patient had an abnormal aortic arch with tortuous arteries that complicated neurointerventional procedures. Cases 1, 2, and 3 underwent computed tomography angiography; case 4 underwent magnetic resonance angiography because the patient was allergic to iodine contrast media.

In this section, we report the clinical histories and interventional procedures for 4 patients with technically challenging anatomy (Figure 1). All procedures were deemed to be within the scope of routine clinical care.

### Case 1

A 90-year-old patient with a history of atrial fibrillation, hypertension, cardiovascular disease, and melanoma presented to the emergency department (ED) with right-sided weakness and global aphasia. Because of a recent gastrointestinal bleed, the patient was not on anticoagulation for atrial fibrillation. The patient's National Institutes of Health Stroke Scale score in the ED was 16. CTA demonstrated a distal left middle cerebral artery M1 occlusion. The patient was taken to Neurointervention for cerebral angiogram and thrombectomy. Because of a markedly tortuous type III aortic arch and a tortuous left carotid artery, the left distal M1 occlusion could not be safely accessed. Despite the use of multiple wires and catheter systems, the intervention was unsuccessful. No further interventions were performed, and the patient elected hospice care. The patient had died by the time of manuscript preparation.

### Case 2

A 76-year-old patient with a history of atrial fibrillation, coronary artery bypass graft, and hyperlipidemia presented to the ED with sudden-onset confusion, dysarthria, and numbness on the right side, accompanied by right upper extremity drift. CTA revealed occlusion in the P1 segment of the left posterior cerebral artery, confirmed by MRI and indicating early ischemic changes. Thrombectomy was scheduled, and attempts were made to access the vertebral arteries through a right common femoral approach. However, navigation through the aortic arch-subclavian anatomy was limited by tortuous origins and the complex course of bilateral vertebral arteries. Despite multiple attempts, including switching catheters and attempting access through the right brachial system, the thrombectomy was unsuccessful in achieving clinically significant recanalization of the P1 segment of the left posterior cerebral artery. Following the procedure, the patient developed a right brachial hematoma without pseudoaneurysm formation. The patient was started on aspirin, was monitored in the neuro intensive care unit, and made a steady recovery, including improvements in visual deficits and pain from the hematoma. The patient was discharged 2 days after admission and transitioned to apixaban from aspirin as an outpatient on day 7.

### Case 3

An 82-year-old patient with a history of heart failure, hyperlipidemia, and hypertension presented with left facial droop, slurred speech, and increased left-sided weakness. Based on suspicion of an acute left middle cerebral artery stroke, cerebral angiogram and mechanical thrombectomy were performed. Despite multiple attempts using left femoral and right brachial access, the carotid arteries and intracranial circulation could not be safely accessed because of a markedly tortuous type III aortic arch, resulting in the decision to refrain from further intracranial interventions. The patient's hospital course was complicated by sepsis, electrolyte imbalances, fluid overload, and worsening respiratory function that required prolonged ventilation. The patient was discharged to a long-term acute care facility 24 days after admission with a tracheostomy and percutaneous endoscopic gastrostomy tubes in place. The patient died a few days after discharge from septic shock.

### Case 4

A 79-year-old patient with no significant medical history was taken to the ED after collapsing at home. Neurologic examination revealed dense hemiparesis and neglect on the left side. Head computed tomography scans showed acute parenchymal and subarachnoid hemorrhage in the right parietal and temporal regions secondary to a suspected ruptured right middle cerebral artery bifurcation aneurysm. Because of the patient's allergy to iodine contrast media, magnetic resonance angiogram (MRA) of the neck was performed instead of CTA. Cerebral angiogram and potential endovascular coil embolization of the ruptured aneurysm were planned. Multiple attempts were made to access the intracranial circulation using catheters introduced through the right femoral artery sheath. However, the patient had a tortuous type III aortic arch, and the femoral approach could not be safely performed. Ultimately, the right radial artery was accessed via the radial approach, allowing for successful angiography and coil embolization of the ruptured aneurysm. The patient remained neurologically stable throughout recovery and was discharged to rehabilitation on postoperative day 22.

## METHODS

### Patient Selection and Ethical Approval

We selected the 4 cases described in the prior section for the fabrication of 3D-printed patient-specific vasculature because of the tortuous aortic arch anatomy that had posed challenges for establishing endovascular access.

The Ochsner Clinic Foundation Institutional Review Board (IRB) granted approval for the use of patient radiographic imaging to create the 3D-printed models (IRB# STUDY00000589). All procedures were in accordance with the ethical standards of the Ochsner Clinic Foundation research committee and with the Declaration of Helsinki and its amendments.

### Fabrication of 3D Models

We used CTA scans of the head, neck, and chest for cases 1, 2, and 3 to capture detailed vascular anatomy. For case 4, we used the MRA of the neck. To create advanced visualization and 3D-printed models, patients’ imaging data files were obtained in DICOM (Digital Imaging and Communications in Medicine) format from the PACS (Picture Archiving and Communication System) in the Ochsner Health Department of Radiology. The DICOM images were acquired through Vertex software (Sorna Corporation) and then exported to the 3D processing software, Mimics Innovation Suite 26 (Materialise NV).

**Figure 2.**
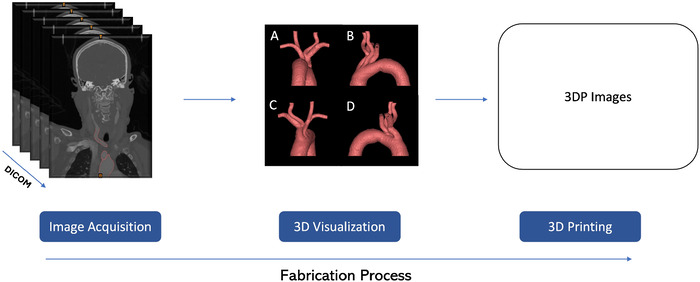
To fabricate the 3D-printed (3DP) patient-specific aortic arches, DICOM (Digital Imaging and Communications in Medicine) images were obtained from the Ochsner Health Department of Radiology PACS (Picture Archiving and Communication System). The images were processed and segmented using computer-aided design software. Four views were captured during the 3D visualization step: (A) anterior, (B) posterior, (C) left, and (D) right. Following segmentation, the extracted regions of interest, referred to as masks, were converted into stereolithography files. These files were used to print the models on a stereolithography 3D printer using flexible resin.

The 3D intracranial vascular model was segmented from the origin of the aorta to below the circle of Willis, marking the internal carotid arteries. Stereolithography files were generated from the segmented aortic arches. For the aortic arches, the solid vascular models were converted into hollow models by postprocessing steps, namely surface smoothing with a factor of 0.7 and hollowing the vascular lumen to a thickness of 1 mm to replicate a vascular wall. The stereolithography files were used to 3D print the models using Flexible 80A Resin (Formlabs) on a Form 3B+ (Formlabs) stereolithography 3D printer. Steps in the fabrication process are shown in Figure 2.

The models were washed in 99% isopropyl alcohol and cured in a heated ultraviolet light chamber at 60 °C for 10 minutes to postprocess the hollow silicone-like vascular model. All 3 branches from the aortic arch, including the vertebral and common carotid arteries, were fabricated.

## RESULTS

**Figure 3.**
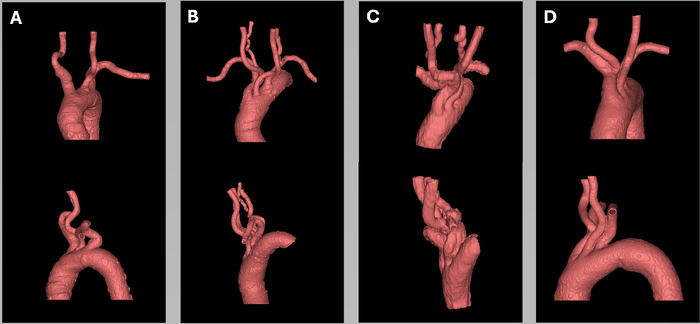
Advanced visualization 3D models of aortic arches that were generated after segmentation from the DICOM (Digital Imaging and Communications in Medicine) images highlight the distinct anatomic features and variations of the aortic arches. The top row shows coronal views, and the bottom row shows sagittal views for (A) case 1, (B) case 2, (C) case 3, and (D) case 4.

**Figure 4.**
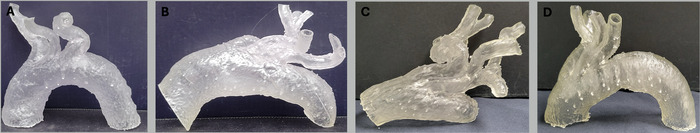
Models of patient-specific type III aortic arches were 3D printed using flexible resin and stereolithography: (A) case 1, (B) case 2, (C) case 3, and (D) case 4. The diameters of major vessels, including the ascending and descending aortic trunks, brachiocephalic, subclavian, common carotid, and vertebral arteries, were measured for all 4 models.

The 3D advanced visualization models generated from the segmented DICOM images are shown in Figure 3. The 3D-printed models depicting the tortuous type III aortic arches are shown in Figure 4. Diameters of major vessels—the ascending and descending aortic trunks and the brachiocephalic, subclavian, and common carotid arteries—were measured for all 4 models (Table). Vertebral arteries were measured for 2 models. The average diameters of the ascending and descending aortic trunks were 33.64 mm and 26.90 mm, respectively. For the brachiocephalic, subclavian, common carotid, and vertebral arteries, the average diameters were 14.22 mm, 7.57 mm, 7.92 mm, and 4.73 mm, respectively. These measured diameters closely match the values reported in the literature, confirming the physical accuracy of the 3D models.^21-27^ Creating an individual anatomic model required an average of 4 hours of digital processing and 13.71 hours of 3D printing, with a materials cost of approximately $17.31.

**Table.** Diameters (in mm) of Major Vessels in 3D-Printed Patient-Specific Models

Arteries		Case 1	Case 2	Case 3	Case 4	Average
Ascending aortic trunk		36.76	37.83	33.00	26.96	33.64
Descending aortic trunk		29.72	30.52	23.00	24.35	26.90
Brachiocephalic artery		17.11	15.63	11.65	12.49	14.22
Subclavian arteries	Left	7.89	8.83	6.19	7.62	7.63
	Right	7.13	8.59	6.67	7.62	7.50
Common carotid arteries	Left	7.90	9.66	7.08	7.72	8.09
	Right	9.20	8.07	6.36	7.36	7.75
Vertebral arteries	Left	Not measured	Not measured	5.58	5.48	5.53
	Right	Not measured	Not measured	3.36	4.50	3.93

## DISCUSSION

3D-printed models of aortic arches and carotid arteries enhance physicians’ perception and understanding of patient-specific anatomy. These models accurately depict the type of aortic arch and the angle between the common carotid and internal carotid arteries.^17^ This angle is associated with a higher risk of stroke or death when ≥60°,^28^ so the ability to measure this anatomic feature can help physicians anticipate challenges and improve procedural planning.^17^ Moreover, 3D-printed models offer better insight into tortuous vasculature than conventional imaging techniques such as CTA and MRA that may not fully capture the 3D complexity of vascular structures.

As previously explained, for this retrospective study, the 3D-printed models were created after the patients had already been treated. All 4 patients had tortuous aortic arch anatomy that hindered access to the intracranial circulation through the conventional femoral approach. As noted in the Clinical Histories section, 3 procedures were unsuccessful via the femoral route, and 1 procedure (case 4) highlights the variability in procedural success based on the access route. Although the intervention in case 4 was eventually performed successfully via the radial approach, multiple attempts to navigate the intracranial circulation through the femoral artery failed because of the patient's anatomy. All 4 cases demonstrate the potential of 3D-printed models to inform preprocedural planning and potentially improve procedural outcomes, regardless of the access route chosen.

Several study limitations must be acknowledged. The 3D-printed models were created for only 4 clinical cases that may not be representative of the full spectrum of anatomic variations and complexities encountered in endovascular procedures. The impact of the 3D-printed models on the patients’ procedural outcomes cannot be assessed because the models were created after the patients were treated. The 3D-printed models may not accurately replicate the mechanical properties of actual blood vessels, such as elasticity and compliance, potentially affecting in vivo application. Technological constraints, such as limitations in 3D-printing resolution and accuracy and in the capabilities of imaging software, may also have an impact on the precision of the anatomic details. Our focus on specific anatomic anomalies limits the generalizability of the findings to all types of cerebrovascular conditions, and the extent to which skills acquired using these models transfer to actual clinical practice remains uncertain.

### Future Directions

**Figure 5.**
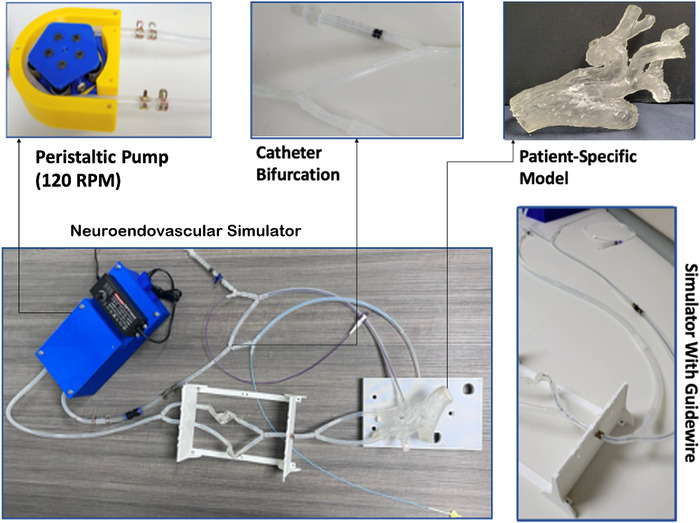
This prototype of a multicomponent patient-specific neuroendovascular simulator includes a peristaltic pump with a rotation speed of 120 revolutions per minute (RPM) that facilitates fluid flow through the circuit. Silicone tubing represents the vascular bifurcation. A 3D-printed patient-specific aortic arch model is connected to the simulator system. A guidewire passing through the simulated vasculature completes the intracranial vasculature-focused circuit.

Neuroendovascular simulators created through 3D printing can be used by trainees to practice procedures that are complicated by complex vascular anatomies and to become familiar with the manipulation of catheters and wires.^15^ We developed a neuroendovascular simulator using the 3D-printed patient-specific aortic model developed for case 3 (Figure 5). For initial testing, the model was integrated into a flow-simulator platform that consisted of a peristaltic pump that was custom-built using a stepper motor, bearings, tubing, and 3D-printed components, controlled by an Arduino, that circulated water through a system of silicone tubes; the 3D-printed model; and a port for the simulated Seldinger technique. The Seldinger technique involves puncturing a vessel with a needle, introducing a guidewire through the needle, and replacing the needle with a sheath or catheter over the guidewire.

The neuroendovascular simulator required 64 hours and 59 minutes of 3D printing and an additional $156.76 in materials and component costs.

Next steps are to incorporate physiologic variables and simulate both laminar and turbulent blood flow rates in the neuroendovascular simulator to replicate dynamic blood flow conditions. We also aim to obtain both computational and physical validation of our models.

## CONCLUSION

This study demonstrated the feasibility of developing low-cost, 3D-printed patient-specific models of the aortic arch and major arteries. These models accurately replicate complex vascular anatomy and are a valuable tool for improving procedural planning and providing physician training in cases involving challenging vasculature.

## Notes

*This article meets the Accreditation Council for Graduate Medical Education and the American Board of Medical Specialties Maintenance of Certification competencies for Patient Care, Medical Knowledge, and Systems-Based Practice.*
